# Characterization of HZO Films Fabricated by Co-Plasma Atomic Layer Deposition for Ferroelectric Memory Applications

**DOI:** 10.3390/nano14221801

**Published:** 2024-11-10

**Authors:** Won-Ji Park, Ha-Jung Kim, Joung-Ho Lee, Jong-Hwan Kim, Sae-Hoon Uhm, So-Won Kim, Hee-Chul Lee

**Affiliations:** 1 Department of Advanced Materials Engineering, Tech University of Korea, Siheung 15073, Republic of Korea; wonji1221@tukorea.ac.kr (W.-J.P.); khj200059@tukorea.ac.kr (H.-J.K.); rmfladl91@tukorea.ac.kr (J.-H.K.); 2 Korea Evaluation Institute of Industrial Technology, Seoul 06152, Republic of Korea; plasma@keit.re.kr; 3 EN2CORE Technology Inc., Daejeon 18469, Republic of Korea; shuhm@en2core.com

**Keywords:** HZO, plasma-enhanced atomic layer deposition, co-plasma, remote plasma system, ferroelectric memory

## Abstract

Plasma-enhanced atomic layer deposition (ALD) is a common method for fabricating Hf_0.5_Zr_0.5_O_2_ (HZO) ferroelectric thin films that can be performed using direct-plasma (DP) and remote-plasma (RP) methods. This study proposed co-plasma ALD (CPALD), where DPALD and RPALD are applied simultaneously. HZO films fabricated using this method showed wake-up-free polarization properties, no anti-ferroelectricity, and high fatigue endurance when DPALD and RPALD started simultaneously. To minimize defects in the film that could negatively affect the low polarization properties and fatigue endurance, the direct plasma power was reduced to 75 W. Thus, excellent fatigue endurance for at least 10^9^ cycles was obtained under a high total remanent polarization of 47.3 μC/cm^2^ and an applied voltage of 2.5 V. X-ray photoelectron spectroscopy and transmission electron microscopy were used to investigate the mechanisms responsible for these properties. The HZO films fabricated by CPALD contained few lattice defects (such as nonstoichiometric hafnium, nonlattice oxygen, and residual carbon) and no paraelectric phase (m-phase). This was attributed to the low-carbon residuals in the film, as high-energy activated radicals were supplied by the adsorbed precursors during film formation. This facilitated a smooth transition to the o-phase during heat treatment, which possessed ferroelectric properties.

## 1. Introduction

Ferroelectric memory is attracting attention as next-generation storage for the AI age, owing to its low power requirements, high speed, and nonvolatility. In particular, Hf_x_Zr_1−x_O_2_ (HZO) films, which consist of HfO_2_-based films doped with Zr, maintain their ferroelectric properties at thicknesses of several nanometers and have excellent compatibility with complementary metal-oxide-semiconductor (CMOS) processes [1,2,3]. HZO films can have different crystal structures, including monoclinic (P21/c, m-phase), tetragonal (P42/nmc, t-phase), and orthorhombic (Pca21, o-phase) phases, depending on the doping composition, grain size, top electrode, deposition process, and heat-treatment conditions, and these structures can produce paraelectric, anti-ferroelectric, and ferroelectric properties, respectively [4,5,6,7,8,9,10,11,12]. To utilize HZO films in next-generation storage, they must have a high remanent polarization (2P_r_), which can be increased by increasing the proportion of the o-phase in the film; excellent fatigue endurance; and low current leakage, which can be reduced by minimizing lattice defects [13,14,15,16].

HZO films with excellent properties can be fabricated using atomic layer deposition (ALD), which can produce very thin uniform films. ALD can be divided into two categories according to the energy source: thermal ALD and plasma-enhanced ALD (PEALD). PEALD is generally preferred, owing to its speed and low deposition temperatures [17,18]. PEALD can be divided into direct-plasma (DP) and remote-plasma (RP) ALD, according to the energy supply method and the position at which the plasma is generated. In DPALD, plasma is generated inside a chamber and directly exposed to the substrate. It can be used to deposit films at low temperatures because it increases the reactivity of the precursors and expands the processable window using the high ion and radical flux of the reactant gas. However, DPALD can cause plasma damage on the substrate, which results in defects that degrade the electrical properties of the film [19,20,21,22,23]. In RPALD, a separate space is used for plasma generation and deposition, and highly reactive radicals are supplied by a remote plasma system (RPS) [24,25,26]. However, according to a previous study conducted by our research group, HZO films deposited using RPALD show anti-ferroelectric properties and a low 2P_r_ value. Furthermore, the radicals generated by the RPS have a low probability of surviving until they reach the substrate, which reduces the growth rate of the film, increases the discharge duration, and increases the processing time [27].

Therefore, this study considered co-plasma ALD (CPALD), where DPALD and RPALD are performed simultaneously. HfO_2_ and ZrO_2_ were deposited at a 1:1 ratio to create 10 nm-thick HZO films. The properties of HZO films fabricated by CPALD and DPALD were compared. Moreover, the effects of various processing conditions, such as whether DP and RP stopped or started at the same time, on the electrical properties of the HZO film, including the polarization–electric field (P–E) hysteresis curve and fatigue endurance, were investigated. Finally, the HZO films fabricated by CPALD and DPALD were analyzed using X-ray photoelectron spectroscopy (XPS) and transmission electron microscopy (TEM) to explore the reasons for the excellent properties of the HZO films.

## 2. Materials and Methods

### 2.1. Fabrication of HZO Films by PEALD

**Figure 1:**
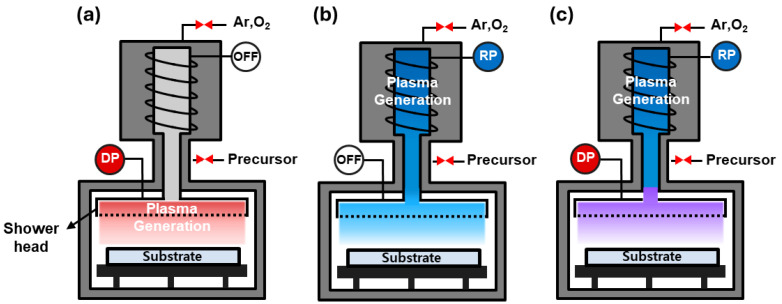
Schematics of plasma power supplies used in this study. Equipment used for (**a**) DPALD, (**b**) RPALD, and (**c**) CPALD.

A 50 nm TiN bottom electrode was deposited on a SiO_2_ (100 nm)/Si (p-type) wafer and used as a substrate for the deposition of HZO films. The Si wafer had a resistivity of 1–30 Ω·cm. Schematics of the equipment used to deposit the HZO films in this study are shown in Figure 1. A remote plasma system (RPS; En2ra-RPS, EN2CORE Technology, Daejeon, Republic of Korea) was also installed on the conventional DPALD equipment (iOV-dx2, iSAC Research, Daejeon, Republic of Korea), which generated plasma in a chamber. Figure 1a shows the DPALD equipment, where the plasma was generated by supplying power only to the electrode inside the chamber. Figure 1b shows the RPALD equipment, where power was only supplied to the RPS, and the electrode inside the chamber was turned off. The radicals generated in the RPS passed through the shower head and were supplied to the inside of the chamber. Figure 1c shows the CPALD equipment, where power was supplied to the electrode inside the chamber and the RPS simultaneously. Tetrakis(ethylmethylamino) hafnium (TEMAH; iChems, Hwaseong, Republic of Korea) and tetrakis(ethylmethylamino)-zirconium (TEMAZ; iChems, Hwaseong, Republic of Korea) were used as the ALD precursors for HfO_2_ and ZrO_2_, respectively [28]. Based on the deposition conditions for single films of HfO_2_ and ZrO_2_, HZO films were deposited with a target thickness of 10 nm and a 1:1 deposition ratio.

**Figure 2:**
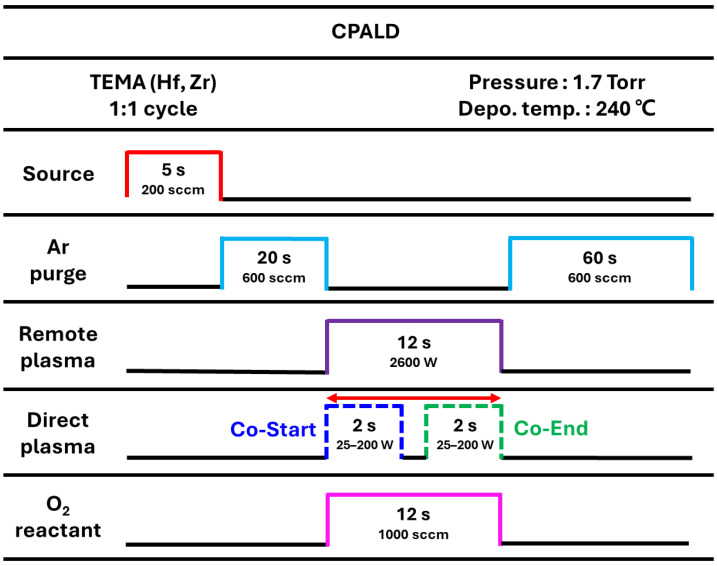
Diagram showing one cycle of CPALD used to fabricate the HZO film. Two procedures, co-start and co-end, were used. The time durations are not plotted to scale.

Figure 2 shows the conditions used to fabricate the HZO film by CPALD. The supply flow rate and duration for the alternating TEMAH and TEMAZ precursors were set to 200 sccm and 5 s, respectively. The radio-frequency (RF) power of the DP inside the chamber was maintained at 25–200 W for 2 s. For the RP, high-density inductively coupled plasma was ignited with a power of 2.6 kW in an Ar atmosphere. Then, O_2_ gas was injected to supply O_2_ radicals to the substrate. For CPALD, the DP and RP were activated simultaneously in the reactant step. CPALD was performed using two procedures, co-start and co-end, where the DP inside the chamber and RP outside the chamber were simultaneously powered on or off, respectively. To fabricate a metal–ferroelectric–metal (MFM) device with a TiN–HZO–TiN structure to evaluate the electrical properties, a dot-shaped TiN top electrode with a diameter of 200 μm and thickness of 50 nm was deposited using lift-off patterning and RF magnetron sputtering. Finally, the HZO film with the top electrode was crystallized by rapid thermal annealing (RTA) for 30 s at 650 °C [27].

### 2.2. Evaluation of the Properties of the PEALD HZO Film

The thickness and refractive index of the HfO_2_ single film were measured using an ellipsometer (Elli-SE, Ellipso Technology, Suwon, Republic of Korea). The crystallinity of the HZO film after heat treatment was evaluated using high-resolution X-ray diffraction (HR-XRD; Smartlab, Rigaku, Tokyo, Japan). The cross-sectional image, thickness, and crystallinity of the HZO films deposited using PALD and DPALD were measured using field-emission transmission electron microscopy (FE-TEM; JEM-2100F, JEOL, Tokyo, Japan). The chemical bonding conditions and ratios of the HZO films were determined using XPS (AXIS-NOVA, Kratos Analytical, Manchester, UK). The electrical properties of the thin films were investigated by measuring the P–E hysteresis curves and fatigue endurance by connecting a microprobe station (APX-6B, WIT, Suwon, Republic of Korea) to a semiconductor measurement system (4200A-SCS, Keithley, Cleveland, OH, USA). The P–E hysteresis loop curve was measured using a ±3 V triangle pulse at a frequency of 1 kHz. In HZO films, cycling the applied field pulse causes the internal oxygen vacancies and atoms to align, which results in a wake-up effect that exhibits stable polarization properties [29,30]. Therefore, the P–E hysteresis loop was measured twice, before wake-up and after 10^4^ cycles, and the results were compared. The fatigue endurance was measured up to 10^9^ cycles with a ±2.5–3 V square pulse at a frequency of 10 kHz [31,32]. 

## 3. Results and Discussion

**Figure 3:**
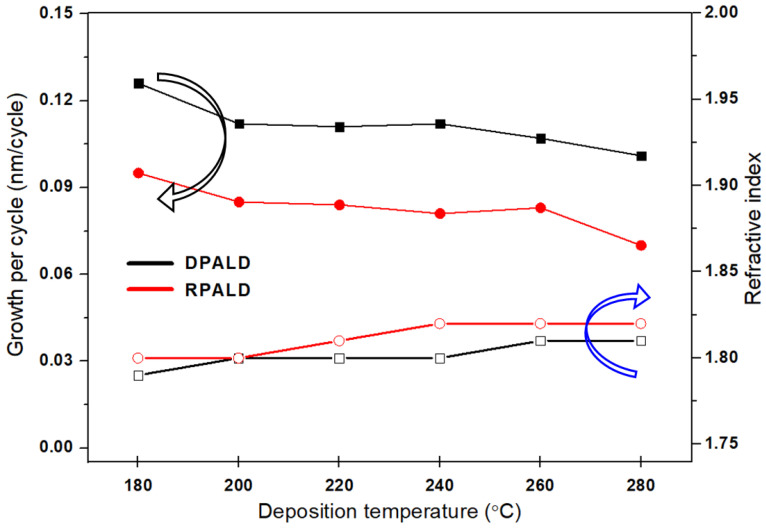
Changes in the GPC and refractive index of the films fabricated by DPALD and RPALD at different deposition temperatures.

A graph of the change in growth per cycle (GPC) and refractive index of the HfO_2_ single films deposited using DPALD and RPALD at 180–280 °C are shown in Figure 3. Both films showed a relatively stable GPC between 200 and 260 °C [33,34]. At a low temperature of 180 °C, the precursor did not react with the reactants and accumulated on the film, which resulted in a relatively high GPC. Conversely, at a high temperature of 280 °C, some of the adsorbed precursors desorbed before a reaction occurred, which resulted in a relatively low GPC. The GPC of the HZO film deposited by DPALD (0.106 nm/cycle) was higher than that of the HfO_2_ film deposited by RPALD (0.077 nm/cycle). This indicates that plasma discharged within the substrate supplied energy for thin film formation more efficiently than plasma supplied from external sources. The refractive index increased slightly as the temperature increased, although no significant change was observed. Therefore, the optimum substrate temperature for depositing HZO composite thin films was 240 °C, the median value of the ALD window.

**Figure 4:**
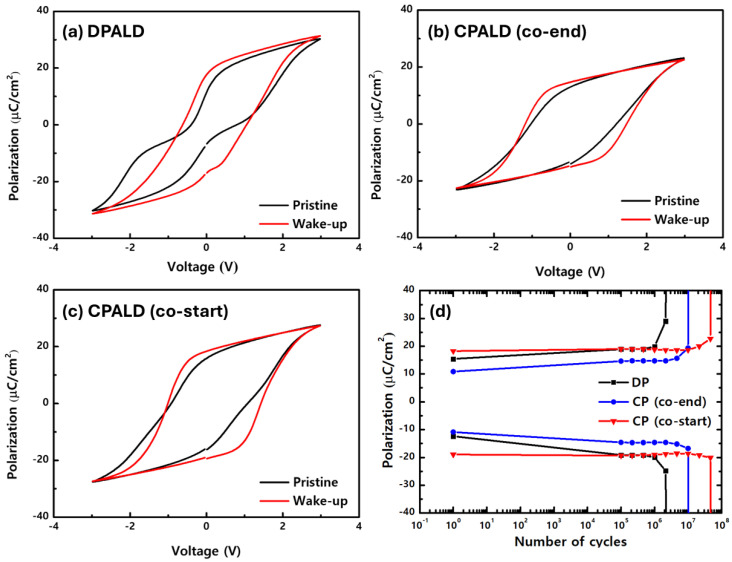
Changes in the P–E hysteresis curves of the HZO films fabricated by (**a**) DPALD, (**b**) CPALD with the co-end procedure, and (**c**) CPALD with the co-start procedure before and after wake-up. (**d**) Comparison of the fatigue endurance of the three HZO films.

Three types of HZO films were fabricated by DPALD, CPALD with the co-end procedure, and CPALD with the co-start procedure at a deposition temperature of 240 °C. Figure 4a–c shows the P–E hysteresis curves for the films before and after wake-up. As shown in Figure 4a, the HZO film fabricated by DPALD exhibited anti-ferroelectricity before wake-up, whereas it showed ferroelectricity after wake-up. Moreover, in the wake-up state, it showed a good 2P_r_ of 35.1 μC/cm^2^. As shown in Figure 4b, the HZO film fabricated by CPALD with the co-end procedure exhibited wake-up-free properties, which indicated that it had ferroelectricity in the pristine state. However, the 2P_r_ in the wake-up state was only 29.9 μC/cm^2^. As shown in Figure 4c, the HZO film fabricated by CPALD with the co-start procedure also exhibited wake-up-free properties. Moreover, in the wake-up state, it showed the best polarization properties, with a 2P_r_ of 39.3 μC/cm^2^.

Figure 4d shows the fatigue endurance of the three HZO films when they were subjected to pulses at a frequency of 10 kHz with an electric field of ±3V for up to 10^8^ cycles. The HZO fabricated by CPALD with the co-start procedure exhibited the highest durability after 4.5 × 10^7^ cycles. The HZO film fabricated by CPALD with the co-end procedure showed fatigue endurance for 10^7^ cycles. Finally, the HZO film fabricated by DPALD exhibited a fatigue endurance for 2 × 10^6^ cycles, which made it the least durable. These differences in polarization and fatigue endurance are expected to be determined by the phase composition of the films and the presence of various defects. Moreover, they will affect the overall electrical properties of the thin films.

**Figure 5:**
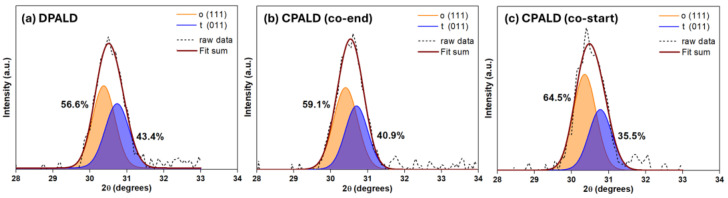
XRD patterns and peak separation results for the HZO films fabricated by (**a**) DPALD, (**b**) CPALD with the co-end procedure, and (**c**) CPALD with the co-start procedure.

The crystallinity of the three HZO films was investigated using XRD and high-resolution XRD measurements were obtained for 2*θ* between 28° and 33°. Peak separation was performed at approximately 30.5° and 30.8°, corresponding to the o- and t-phases, respectively, and the relative ratio between the two phases was determined by combining the peaks and calculating the ratio of the obtained areas. Each peak was separated using the Gaussian deconvolution method, assuming that there was no excessive stress or deformation within the films [35]. The XRD patterns and peak separation results corresponding to each phase are shown in Figure 5. All three HZO films underwent crystallization following RTA at 650 °C for 30 s. The HZO film fabricated by DPALD (Figure 5a) contained a relatively high proportion of the o-phase (56.6%), which exhibited ferroelectricity, compared to the t-phase. However, the films fabricated by CPALD with the co-end and co-start procedures showed even higher proportions of the o-phase (59.4% and 64.5%, respectively). This confirmed that the HZO film fabricated by CPALD with the co-start procedure, which showed the best polarization and fatigue endurance, exhibited the highest o-phase ratio [36,37].

**Figure 6:**
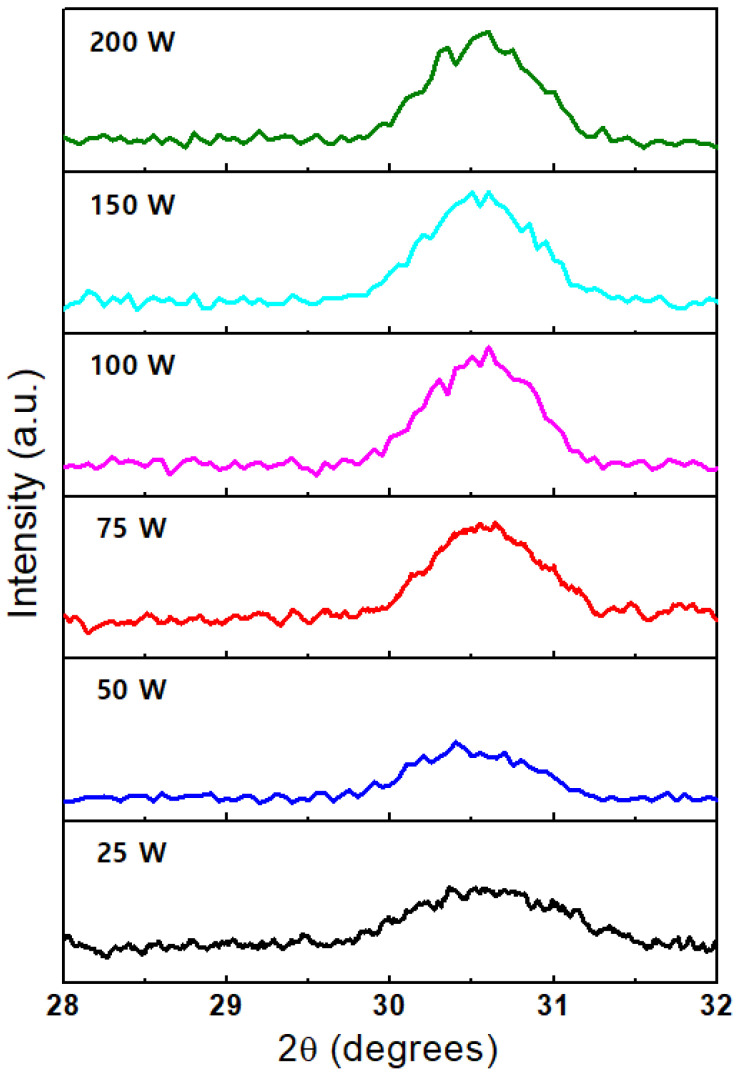
XRD patterns of the HZO films fabricated by CPALD with the co-start procedure using different DP powers.

This study aimed to reduce the RF power of the DP during CPALD because DP can cause defects within the film that can adversely affect polarization and fatigue endurance [38]. Therefore, HZO films were fabricated by CPALD with the co-start procedure (which exhibited superior properties in the previous experiments) and the DP power was varied from 25 to 200 W. The XRD patterns of these films are shown in Figure 6. The thin film deposited at a relatively low DP power of 25 W underwent crystallization following RTA at 650 °C. Furthermore, as the DP power increased, the XRD peak intensity increased until it reached a saturation point at 75 W.

**Figure 7:**
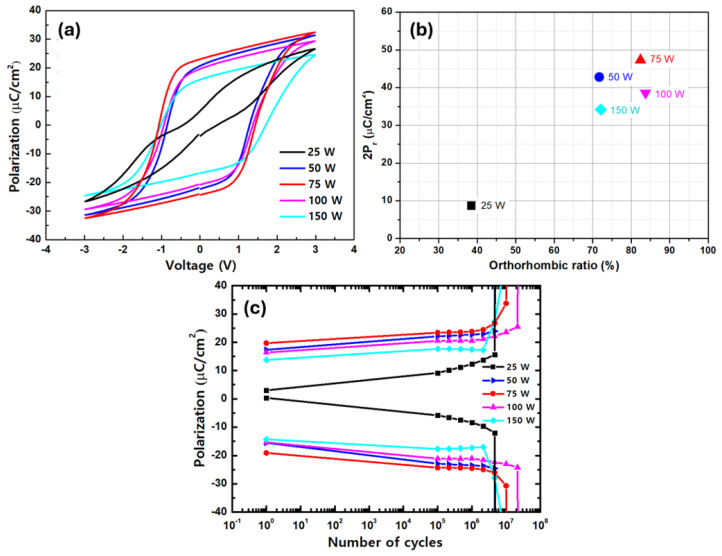
(**a**) P–E hysteresis curves, (**b**) 2P_r_ vs. orthorhombic phase ratio, and (**c**) fatigue endurances of the HZO films fabricated by CPALD with the co-start procedure with different DP powers.

The P–E hysteresis curves of the films in the wake-up state are shown in Figure 7a. The overall 2P_r_ increased and then decreased as the DP power increased. Even in the wake-up state, the film fabricated with a DP power of 25 W showed anti-ferroelectric properties. This was probably because there was insufficient energy during film formation, which prevented the complete transition to the o-phase during RTA [8]. The maximum 2P_r_ value was 47.3 μC/cm^2^ when the DP power was 75 W, whereas it decreased to 38.6 and 34.2 μC/cm^2^ when the DP power increased to 100 and 150 W, respectively.

As shown in Figure 7b, the relationship between the orthorhombic ratio and 2P_r_ at varying DP power is derived from the data presented in Figure 6 and Figure 7a. The two parameters appear to exhibit an almost proportional relationship. In particular, HZO thin films at lower DP powers, with the exception of 25 W, demonstrated higher 2P_r_ values compared to those with similar orthorhombic ratios. This phenomenon can be attributed to the combined effects of reduced plasma damage and an elevated orthorhombic ratio, both of which positively influence the polarization properties of the HZO films

The fatigue endurance of the films under a ±3 V square pulse with a frequency of 10 kHz is shown in Figure 7c. The film deposited with a DP power of 100 W exhibited the highest fatigue endurance of 2.1 × 10^7^ cycles, whereas the film deposited with a DP power of 75 W, which exhibited the best polarization properties, exhibited the fatigue endurance of 1.1 × 10^7^ cycles.

**Figure 8:**
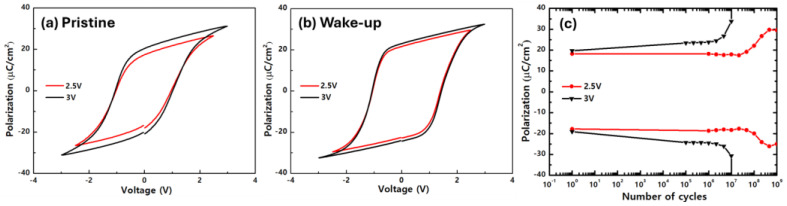
Properties of the HZO film fabricated by CPALD with the co-start procedure, a DP power of 75 W, and measurement voltages of 2.5 and 3 V. P–E hysteresis curves in the (**a**) pristine and (**b**) wake-up states. (**c**) Fatigue endurance up to 10^9^ cycles.

In ferroelectrics, electrical fatigue occurs as defects within a thin film migrate and accumulate in specific areas during polarization reversal [39,40]. The HZO film fabricated by CPALD with the co-start procedure and a DP power of 75 W, which had a maximum 2P_r_ of 47.3 μC/cm^2^, was expected to show a relatively large internal field, owing to its relatively good polarization properties. However, in this case, when the fatigue endurance was measured at the same applied voltage, it could be underestimated owing to the relatively active movement of defects. Figure 8 shows the electrical properties of the HZO film measured at a voltage of 2.5 V, lowered from 3 V. As shown in Figure 8a, even when the measurement voltage was reduced to 2.5 V, there was little change in the polarization properties in the pristine state before wake-up. Moreover, when comparing the polarization properties in the wake-up state, there was no significant difference between the 2P_r_ measured at 3 and 2.5 V (47.3 and 44.3 μC/cm^2^, respectively). Thus, the 2P_r_ measured at 2.5 V in the wake-up state was greater than those measured at 3 V under different DP power conditions (as shown in Figure 7).

The fatigue properties of the HZO film measured at 2.5 V were compared to those previously measured at 3 V. This confirmed that the electrical durability of the thin film was improved, as the HZO film did not break down after 10^9^ cycles. After 10^8^ cycles, the 2P_r_ value significantly increased. This was believed to be owed to the increase in the leakage current caused by the accumulation of defect layers within the thin film, rather than an improvement in the polarization properties.

Comparative XPS analysis was used to investigate the reasons why the HZO film fabricated by CPALD exhibited superior crystallinity and electrical properties compared to that fabricated by DPALD [41]. The HZO film fabricated by CPALD with the co-start procedure and a DP power of 75 W, which exhibited excellent polarization and fatigue endurance, was analyzed. HZO films fabricated by DPALD with DP powers of 75 and 200 W were also analyzed to investigate the effects of the DP power on structural damage.

**Figure 9:**
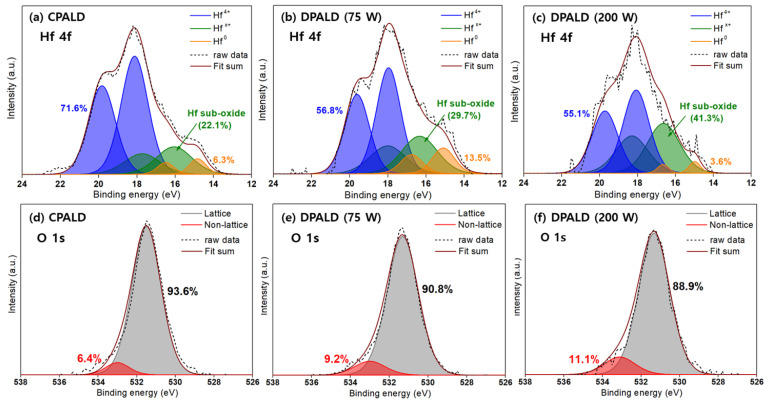
HZO films deposited using CPALD and DPALD methods: (**a**–**c**) Hf 4f and (**d**–**f**) O1s peak XPS narrow scan. The DPALD method was performed in two conditions of 75 and 200 W to verify the effects on the DP power.

Figure 9a–c shows the narrow XPS scan results for the Hf 4f peaks of these HZO films. The two purple peaks on the side with relatively high binding energies corresponded to stoichiometric HfO_2_. The two green peaks with relatively low binding energies corresponded to lattice defects in nonstoichiometric HfO_2−x_. The relative nonstoichiometric HfO_2−x_ ratios, obtained by integrating the areas of the two peaks, for the HZO films fabricated by CPALD and DPALD with a DP power of 75 W were 22.1% and 29.7%, respectively. However, that for the HZO fabricated by DPALD with a DP power of 200 W was 41.3%, which indicates that DP may induce crystallographic defects within the film.

Figure 9d–e shows the narrow XPS scan results for the O 1s peaks of the three HZO films. These O 1s peaks can be deconvolved into peaks corresponding to oxygen bonding in a perfect crystal and non-lattice peaks such as oxygen vacancies, O–H, and C–O. In this case, the non-lattice peaks appeared 1.4–1.6 eV away from the lattice peaks [42]. The HZO film produced by CPALD exhibited the lowest non-lattice oxygen ratio of 6.4%, which was consistent with previous results for Hf. The HZO films produced by DPALD with DP powers of 75 and 200 W had ratios of 9.2% and 11.1%, respectively. This is thought to be because the supply of activated oxygen radicals can easily reduce the carbon content during the reaction stage, which mitigates the damage to the film caused by the DP power [43]. When comparing the C 1s content of the films, the HZO film produced by CPALD and DPALD with a DP power of 75 W had ratios of 1.2% and 1.8%, respectively.

**Figure 10:**
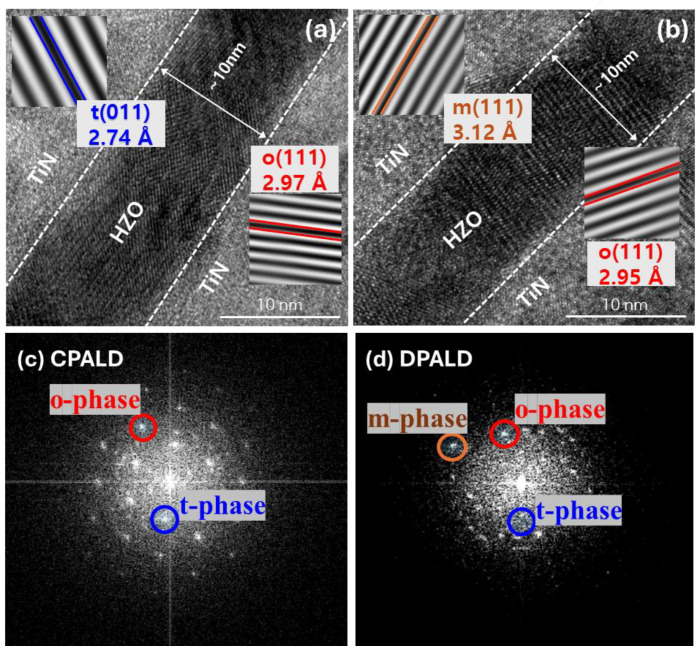
Cross-sectional HR-TEM images of HZO films fabricated by (**a**) CPALD and (**b**) DPALD. FFT patterns of HZO films fabricated by (**c**) CPALD and (**d**) DPALD.

Figure 10 shows high-resolution TEM (HR-TEM) cross-sectional images and fast Fourier transform (FFT) patterns of the HZO films fabricated by CPALD and DPALD. As shown in Figure 10a,b, the MFM structures of the TiN–HZO–TiN thin films were well-formed, with relatively distinctive interfaces. All the films were fabricated using the super-cycle method, with the ratio of HfO_2_ and ZrO_2_ adjusted to 1:1, for a total of 47 cycles. This resulted in thin films with a thickness of approximately 10 nm, regardless of the fabrication conditions. When the HR-TEM cross-sectional images were considered in combination with the FFT patterns in Figure 10c,d, the results showed that sufficient crystallization occurred in all the thin films. In the case of the HZO film fabricated by CPALD, only the o- and t-phases with d-spacings of 2.97 and 2.74 Å, respectively, were observed. By contrast, in the case of the HZO film fabricated by DPALD, the m-phase with a d-spacing of 3.12 Å was also observed [44]. CPALD supplies high-energy radicals during the reactant stage, which can remove the carbon component, thereby alleviating defects caused by DP. Thus, this lattice state inhibits the transition to the m-phase.

## 4. Conclusions

In this study, HZO ferroelectric films with excellent electrical properties and high o-phase ratios were fabricated by CPALD, wherein DPALD and RPALD are performed simultaneously. The HZO film fabricated by CPALD, optimized by reducing the DP power to 75 W and using the co-start procedure, exhibited a high 2P_r_ of 47.3 µC/cm^2^ and excellent fatigue endurance for more than 10^9^ cycles at 2.5 V. XRD, XPS, and TEM analyses showed that the HZO film fabricated by CPALD did not contain the m-phase, and the proportion of the o-phase, which exhibits ferroelectricity, was relatively high. Furthermore, the ratio of nonstoichiometric Hf and non-lattice oxygen within the thin film was low. These results are consistent with the electrical properties of the HZO film. Because CPALD can remove carbon components and mitigate plasma damage by supplying high-energy radicals during the formation of thin films, it is expected to be suitable for other ALD applications.

As a result, we confirmed a new thin film deposition process utilizing DP and RP simultaneously, which has not been previously studied. This process can improve the electrical performance, reduce fatigue degradation caused by plasma damage, and resolve throughput issues, thereby enhancing the properties of HZO ferroelectric thin films for next-generation memory applications.

## Data Availability

The data presented in this study are contained within the article.
